# Transmissibility of Syphilis

**Published:** 1891-09

**Authors:** 


### Transmissibility of Syphilis.

As published in Prof. Morrow's *Atlas of Venereal and Skin Diseases*, conclusions in reference to the hereditary transmissions of syphilis are as follows :

1. A syphilitic man may beget a syphilitic child, the mother remaining exempt from all visible signs of the disease ; the transmissive power of the father is comparatively restricted.

2. A syphilitic woman may bring forth a syphilitic child, the father being perfectly healthy ; the transmissive power of the mother being much more potent and pronounced and of longer duration than that of the father. When both parents are syphilitic, or the mother alone, and the disease recently acquired, the infection of the fœtus is almost inevitable ; the more recent the syphilis the greater the probability of infection, and the graver the manifestation in the offspring.

3. While hereditary transmission is more probable when the parental syphilis is in full activity of manifestation, it may also be effected during a period of latency when no active symptoms are present.

4. Both parents may be healthy at the time of procreation, and the mother may contract syphilis during her pregnancy and infect her child in utero. Contamination of the fœtus during pregnancy is not probable if the maternal infection takes place after the seventh month of pregnancy.

